# From genomics to treatment: overcoming pan-drug-resistant *Klebsiella pneumoniae* in clinical settings

**DOI:** 10.3389/fphar.2025.1570278

**Published:** 2025-05-30

**Authors:** Fernando Pasteran, Juan Manuel De Mendieta, Natalia Pujato, Gina Dotta, Lisandro J. González, Mabel Rizzo, Alejandra Fernández, Paola Ceriana, Lucia Maccari, Melina Rapoport, Sonia Gómez, Celeste Lucero, María Alejandra Menocal, Ezequiel Albornoz, Denise De Belder, Marcelo Radisic, Alejandro J. Vila, Alejandra Corso

**Affiliations:** ^1^ Servicio Antimicrobianos, National Reference Laboratory for Antimicrobial Resistance, Instituto Nacional de Enfermedades Infecciosas, ANLIS “Dr. Carlos Malbrán”, Buenos Aires, Argentina; ^2^ Instituto de Trasplante de Alta Complejidad, Buenos Aires, Argentina; ^3^ Instituto de Biología Molecular y Celular de Rosario, CONICET, Universidad Nacional de Rosario, Rosario, Argentina; ^4^ Facultad de Ciencias Bioquímicas y Farmacéuticas, Universidad Nacional de Rosario, Rosario, Argentina; ^5^ Laboratorio de Microbiología, Hospital Interzonal General de Agudos “Dr. José Penna”, Bahía Blanca, Argentina; ^6^ Laboratorio de Microbiología “Dr. Rapela”, Buenos Aires, Argentina; ^7^ Unidad Operativa Centro Nacional de Genómica y Bioinformática, Instituto Nacional de Enfermedades Infecciosas, ANLIS “Dr. Carlos Malbrán”, Buenos Aires, Argentina

**Keywords:** multi-drug resistance, emerging pathogens, *Klebsiella pneumoniae*, pan-drug resistance, aztreonam-avibactam, NDM carbapenemase, cefiderocol, cefepime-zidebactam

## Abstract

**Introduction:**

The spread pan-drug resistant pathogens pose a critical challenge to current therapies, resulting in high mortality and necessitating alternative approaches.

**Methods:**

We report pan-drug resistant Klebsiella pneumoniae isolates from five patients in a single hospital, including resistance to cefiderocol and cefepime-zidebactam in one isolate.

**Results:**

Whole-genome sequencing identified blaNDM-5 and blaCTX-M-15 genes in all isolates, explaining carbapenemase and extended-spectrum β- lactamase phenotypes, with blaKPC-2 in one isolate. A novel sulfhydryl variable β-lactamase (SHV) variant, blaSHV-231, was present in all isolates under a strong promoter. Two isolates exhibited a non-synonymous mutation in fstI encoding PBP3, the primary target of aztreonam in Gram-negative bacteria. Genomic and phenotypic characterization guided successful compassionate treatment using aztreonam, ceftazidime-avibactam, and amoxicillin-clavulanate at maximum doses.

**Discussion:**

Dissection of the roles of the substitutions present in blaSHV-231 revealed that this variant was responsible for the reduced susceptibility to aztreonam-avibactam, at the expense of a higher susceptibility to clavulanate. Targeted therapy can be successful upon dissection of unexpected mechanisms of resistance that enhance the contribution of endemic β-lactamase.

## 1 Introduction

Antimicrobial resistance is a critical global threat to human health. The emergence of multidrug resistance in Gram-negative pathogens has outpaced the development, distribution and availability of new antimicrobial agents (Bonomo et al., 2024). Particularly concerning is the recent rise of pan-drug-resistant (PDR) Gram-negative bacteria, which are non-susceptible to all conventional antimicrobial agents (Karakonstantis et al., 2019). PDR infections, predominantly involving *Klebsiella pneumoniae*, *Acinetobacter baumannii* and *Pseudomonas aeruginosa* isolates in critically ill patients, are associated with high mortality rates and pose a significant public health threat (Karakonstantis et al., 2019).

The recent development of new antimicrobials to combat carbapenemase-producing organisms, such as ceftazidime-avibactam, alone or in combination with aztreonam, imipenem-relebactam, meropenem-vaborbactam and cefiderocol has provided some relief for treating challenging strains (Yahav et al., 2020; Soman et al., 2024; Wang et al., 2024). However, there are reports of resistance events to last-generation drugs, and the restricted accessibility to some of these drugs in most low- and middle-income countries excludes them from the current consensus definitions of PDR (Magiorakos et al., 2012; Yahav et al., 2020).

Metallo-β-lactamase (MBL) producers, among the most widely disseminated carbapenemase globally, are effectively treated using cefiderocol or a combination therapy of ceftazidime-avibactam and aztreonam (Tamma et al., 2024; Wise et al., 2024). In this combination, aztreonam evades hydrolysis by MBLs, while avibactam neutralizes frequently co-produced extended-spectrum β-lactamases (ESBLs). This allows aztreonam to effectively reach its target site, the penicillin-binding protein 3 (PBP3) (Georgopapadakou et al., 1982; Yahav et al., 2020).

Carbapenemase producers are endemic in Argentina, with *bla*_NDM_ and *bla*_KPC_ being responsible for 88% of carbapenemase-producing Enterobacterales infections (Echegorry et al., 2024). NDM producers in Argentina are typically susceptible to aztreonam plus ceftazidime-avibactam (Echegorry et al., 2024). Only one previously documented outbreak of PDR Enterobacterales had occurred in Argentina, before the local approval of ceftazidime-avibactam, driven by expansion of an NDM-1 plus OXA-163-producing *Providencia stuartii* clone (Macchi et al., 2017).

Here, we report the emergence of closely related NDM-producing *K. pneumoniae* clinical isolates that initially exhibited resistance to all available antibiotics, including aztreonam-avibactam and subsequently evolved to resistance to both, cefiderocol and cefepime-zidebactam. Whole-genome sequencing (WGS) revealed the presence of multiple β-lactamases and other critical mutations in the resistome. This resistance phenotype led us to a successful compassionate treatment using maximum doses of aztreonam, ceftazidime-avibactam, and amoxicillin-clavulanate. These isolates demonstrated notable resistance to aztreonam-avibactam, which we attribute to the novel ESBL, SHV-231, responsible of a reduced susceptibility to avibactam at the expense of a higher susceptibility to clavulanate. This shows that the identification of the resistance mechanisms helps dealing with challenging PDR infections by resorting to alternative compassionate therapies.

### 1.1 Clinical case presentation

Five cases of PDR *K. pneumoniae* were identified in a single institution in Buenos Aires City between August 2022 and January 2023. All patients had undergone kidney transplants.

Patient 1: a 38-year-old male with type 1 diabetes developed a urinary tract infection (UTI) from NDM-producing *K. pneumoniae* after a renal-pancreatic transplant. Initial treatment with ceftazidime-avibactam and aztreonam was effective, but an NDM-producing *K. pneumoniae* strain with a PDR phenotype (Kpn-1-M28162) appeared in August 2022, requiring further medical attention. Compassionate treatment with ceftazidime-avibactam and aztreonam infusion, and oral amoxicillin-clavulanate (7 days) led to improved symptoms (the rationale for this triple antibiotic scheme is explained in further sections).

Patient 2: a 38-year-old male developed a UTI from an MBL-producing *K. pneumoniae* strain (PDR phenotype, Kpn-2-M28195) shortly after kidney transplant. Treatment with ceftazidime-avibactam and aztreonam infusion, and oral amoxicillin-clavulanate led to improved symptoms. In February 2023, gastrointestinal colonization by the same PDR germ was confirmed.

Patient 3: a 54-year-old male with chronic renal failure received a kidney transplant in August 2022. Recurrent UTIs following transplantation, including an episode in November caused by an MBL-producing *K. pneumoniae* strain (PDR phenotype, Kpn-3-M28196), was successfully managed with ceftazidime-avibactam and aztreonam. In December, a new UTI episode due to PDR *K. pneumoniae* isolate was compassionate treated with ceftazidime-avibactam and aztreonam infusion, and oral amoxicillin-clavulanate for 7 days that led to improved symptoms. Subsequent urine cultures were negative, and the patient showed favorable progress, leading to discharge.

Patient 4: a 42-year-old male with end-stage renal disease and on hemodialysis since 2016 faced multiple UTIs after a kidney transplant between April and September 2022 due to MBL-producing *K. pneumoniae*, treated with ceftazidime-avibactam and aztreonam or colistin plus rifampin. UTIs recurred in September, October, and November 2022 (PDR phenotype Kpn-4-M28206). The patient received compassionate treatment with ceftazidime-avibactam and aztreonam, and oral amoxicillin-clavulanate for 10 days in October and 4 weeks in December. Throughout, the patient remained afebrile with stable renal function, asymptomatic, and was ultimately discharged.

Patient 5: a 34-year-old female with type 1 diabetes and long-term hemodialysis had a combined kidney-pancreas transplant in late 2022. In February 2023, she presented with a UTI caused by a PDR *K. pneumoniae* strain (Kpn-5-M28413). Treatment was initiated with ceftazidime-avibactam and aztreonam, and amoxicillin-clavulanate and resulted in clinical cure. Her condition further deteriorated due to surgical complications, developing candidemia, coagulopathy, and an upper gastrointestinal hemorrhage, ultimately leading to her death in late February 2023.

## 2 Materials and methods

### 2.1 Bacterial identification and susceptibility testing

Bacterial identification was performed by MALDI-TOF (Bruker, United States) (Clinical and Laboratory Standards Institute, 2017). Susceptibility testing was conducted through agar dilution, disc diffusion and broth microdilution (BMD) using either commercial panels (Sensititre, Thermo-Fisher) or an in-house BMD method, employing either cation-adjusted Mueller-Hinton (CAMHB, Difco, United States) or iron-depleted CAMHB for cefiderocol (Clinical and Laboratory Standards Institute, 2024a; Clinical and Laboratory Standards Institute, 2024b). The panel of agents tested included: ampicillin, cefazolin, cefotaxime, ceftazidime, cefepime, aztreonam, cefoxitin, ertapenem, meropenem, imipenem, amoxicillin-clavulanate, ampicillin-sulbactam, piperacillin-tazobactam, ceftolozane-tazobactam, ceftazidime-avibactam, aztreonam-ceftazidime-avibactam, aztreonam-avibactam, aztreonam-relebactam, imipenem-relebactam, cefepime-zidebactam, gentamicin, amikacin, ciprofloxacin, trimethoprim-sulfamethoxazole, minocycline, tigecycline, chloramphenicol, rifampin, nitrofurantoin, fosfomycin, colistin and cefiderocol.

MICs were interpreted based on the latest CLSI criteria, except for aztreonam-avibactam, colistin and fosfomycin, which followed EUCAST guidelines, and tigecycline, interpreted with FDA standards (U.S. Food and Drug Administration, 2025; Clinical and Laboratory Standards Institute, 2024c; European Committee on Antimicrobial Susceptibility Testing, 2024).

To assess pertinent resistance mechanisms, susceptibility tests were conducted for selected agents by supplementing agar dilution plates with dipicolinic acid (Sigma, United States) at a final concentration of 1,000 μM, serving as an MBL inhibitor (Bahr et al., 2021). Aztreonam potentiation tests involved the same procedure by agar dilution, testing aztreonam alone or with avibactam, relebactam, clavulanic acid, and/or rifampin at a fixed concentration of 4 mg/L, and/or ceftazidime, ertapenem and fosfomycin at a fixed concentration of 10 mg/L, aligned with concentrations defined by PK/PD (Majumdar et al., 2002; Rodríguez-Gascón and Canut-Blasco, 2019; Clinical and Laboratory Standards Institute, 2024c). Zidebactam combinations were tested in a fixed ratio of 1:1 (Clinical and Laboratory Standards Institute, 2024c). Potentiation was defined as a 2-fold decrease in the MIC value of aztreonam in the presence of the inhibitor and/or accompanying antimicrobial agent.

### 2.2 Molecular characterization of the isolates

Initial characterization of resistance mechanisms was carried out using multiplex PCR to detect the most prevalent carbapenemase genes, including *bla*_KPC_, *bla*_NDM_, *bla*_VIM_, *bla*_IMP_, and *bla*_OXA-48_-like. Additional analyses included multiplex PCR for ESBLs (*bla*_CTX-M_, *bla*_PER_) and plasmid-mediated AmpC β-lactamases (*bla*_CMY_) (Faccone et al., 2023).

### 2.3 Conjugation assays

Biparental conjugation assays were conducted on solid media using Kpn-2 and Kpn-4 aztreonam-avibactam-resistant isolates. *Escherichia coli* J53 (azide-resistant and susceptible to aztreonam-avibactam) served as the recipient strain. Donor and recipient strains were mixed in a 3:1 ratio on tryptic soy agar plates and incubated at 35°C for 18 h. The conjugation mix was then resuspended in 1 mL of saline, and transconjugants were selected on tryptic soy agar plates containing 200 mg/L azide and 1 mg/L aztreonam plus 4 mg/L avibactam.

### 2.4 WGS and bioinformatic tools

Genomic DNA was extracted using the QIACube connect system with QIAamp DNA Blood Mini Kit (Qiagen, Germantown, MD, United States). Short-read sequencing was performed using the Nextera XT DNA library preparation kit and the Illumina MiSeq technologies (Illumina, San Diego, CA, United States) to generate 250 bp paired-end reads performed at the Unidad Operativa Centro Nacional de Genómica y Bioinformática ANLIS Malbrán. Quality control and trimming of the reads were performed by FastQC v0.11.5 and Trim Galore v0.6.3, respectively. Reads were *de novo* assembled using Unicycler v0.4.8-beta and taxonomy was confirmed with Kraken2 v2.1.2. AMRfinderPlus v3.11.17 was used to identify resistance genes and Prokka v.1.14 for annotation (Seemann, 2014; Wick et al., 2017; Feldgarden et al., 2020). Additionally, other bioinformatics tools were used to search for or compare specific genes, genomic regions, or amino acid sequences, like BLASTn and UniProt (Bateman et al., 2021). The confirmation of nucleotide sequences for *bla*_SHV_, *bla*_NDM_, and *bla*_KPC_ was conducted through in-house PCR using Sanger technology ABI PRISM 3100 (Applied Biosystems, United States). The PCR reaction was assembled with 0.2 mM of dNTPs, 1.5 mM of MgCl_2_, 1X buffer, and 1.5 U of Taq polymerase (Invitrogen, United States) in a final volume of 50 µL. The primer concentration for *bla*_SHV_, *bla*_NDM_, and *bla*_KPC_ was set at 0.2 µM. The specific primers utilized were as follows: SHV-Fa [5′-GCCCGGTTATTCTTATTTGTCGC-3′] and SHV-Ra [5′-TCTTTCCGATGCCGCCGCCAGTCA] for *bla*_SHV_, NDMin-F [5′-CTATTTACTAGGCCTCGCATT] and NDMin-R [5′-ATAAAACGCCTCTGTCACAT] for *bla*_NDM_, and KPC-F [5′-AACAAGGAATATCGTTGATG-3′] and KPC-R [5′-AGATGATTTTCAGAGCCTTA-3′] for *bla*_KPC_. These primer sets were designed to yield amplicons of 1,016, 936, and 916 bp, respectively.

### 2.5 Phylogenetic analysis

The genetic relationships among isolates were assessed by constructing a pairwise single nucleotide polymorphism (SNP) distance matrix and a phylogenetic tree based on a SNPs core genome alignment. The core genome alignment was first generated using Roary v3.12.0, concatenating all genes present in 99% of the samples. Subsequently, snp-sites v2.5.1 was used to create a core genome alignment consisting solely of SNPs, and snp-dist v0.8.2 was employed to generate the SNP difference matrix. Prevouisly reported SNP cutoffs (less than 21 SNPs) was used to define hospital clusters (David et al., 2019). A maximum likelihood phylogenetic tree was generated using RAxML v8.2.11, employing the GTR model with gamma-distributed site heterogeneity (GTRGAMMA) and 1,000 bootstrap replicates, based on the SNPs core alignment. The resulting tree was visualized using MEGA v11.0.13. Reference isolates include the following NCBI bio projects: NZ_JARERZ010000001, CP006923, CP006918, GCF_000316245.2, AUSMDU00008079, GCF_000599925.1 (Deleo et al., 2014; Venturini et al., 2019). Core genome MLST (cgMLST) analysis was also studied with the PathogenWatch tool based on the allelic profile of 629 conserved genes.

### 2.6 Cloning of *bla*_SHV_ allelic variants and susceptibility testing of recombinant clones

The *bla*_SHV_ variants were engineered by incorporating a Strep Tag sequence at the C-terminal end for immunodetection and cloned into the pMBLe vector, enabling protein expression through IPTG-controlled. *E. coli* DH5α cells were transformed with these vectors using the chemical method and cells were selected with 50 mg/L gentamicin. PCR amplification and Sanger sequencing confirmed the integrity of the synthesized genes. The susceptibility of *E. coli* expressing various *bla*_SHV_ alleles was tested in 100 µM IPTG by measuring the MICs of different antibiotics (González et al., 2016).

### 2.7 Structural modeling of PBP3 mutation

To visualize the potential structural impact of the A413V substitution in PBP3, we performed *in silico* analysis using the PyMOL Molecular Graphics System, Version 3.0 (Schrödinger, LLC). The wild-type and mutant amino acid sequences were modeled based on available structural templates, and the A413V mutation was introduced manually.

### 2.8 PDR definition

The strains were classified as PDR according to the consensus definition established by Latin American countries in coordination with Pan American Health Organization (PAHO/WHO), which defines PDR in Enterobacterales as phenotypic resistance to penicillins, cephalosporins, monobactams, carbapenems, legacy β-lactam/β-lactamase inhibitor combinations, aminoglycosides, fosfomycin, lipopeptides, tetracyclines/glycylcyclines, quinolones and folate pathway inhibitors (Jiménez Pearson et al., 2019). This definition was subsequently expanded at the national level by the Argentine Ministry of Health to include newer antimicrobials incorporated into the national formulary, such as ceftolozane-tazobactam, ceftazidime-avibactam, aztreonam-avibactam, and imipenem-relebactam (https://www.argentina.gob.ar/sites/default/files/2021/08/instructivo_pandrogoresistencia-16092024.pdf). As cefiderocol is not currently listed in the national vademecum, it was initially excluded from the local definition of PDR.

## 3 Results

### 3.1 Identification of PDR *K. pneumoniae* and antimicrobial susceptibility

The five isolates were identified as *K. pneumoniae* by MALDI-TOF. MIC determination confirmed the PDR phenotype in all isolates, including, ceftazidime-avibactam (MIC, >256 mg/L), aztreonam-avibactam or aztreonam-relebactam (32 - >256 mg/L, see details in other section), imipenem-relebactam (>32 mg/L), tigecycline (>2 mg/L), fosfomycin (>64 mg/L), and colistin (>8 mg/L). Susceptibility to cefiderocol was demonstrated in Kpn-1 to Kpn-4 strains (MICs 1–2 mg/L), on the contrary Kpn-5 rendered resistance (>8 mg/L). Cefepime-zidebactam demonstrated promising activity against Kpn-1 and Kpn-2 (8 mg/L). However, Kpn-3 and Kpn-4 exhibited a marked increase in MIC values (32 mg/L), and Kpn-5 showed a high level of resistance to this combination (>64 mg/L). The carbapenem phenotype was abolished by the metal chelator dipicolinic acid, revealing the presence of an MBL, except for Kpn-3, indicating additional mechanisms.

### 3.2 PCR and WGS analysis

Initial characterization of resistance mechanisms by PCR revealed the presence of *bla*_NDM_ and *bla*_CTX-M_ in all isolates, while *bla*_KPC_ was detected exclusively in Kpn-3.

WGS confirmed the taxonomic identity of the *K. pneumoniae* isolates (5,431,038–5,542,594 bp), classified as clonal group 258 (CG258) with capsular type K107/O1/O2V2 (wzi 154). The isolates were genetically related with 31–89 SNPs between them (Table 1). This polymorphism exceeded the threshold typically used to define outbreak clusters, suggesting the circulation and local evolution of a closely-related strains within the institution. Phylogenetic analysis based on maximum likelihood revealed that the isolates belong to Clade II of the ST258 lineage (Supplementary Figure S1). According to cgMLST, all five isolates were close to core genome sequence type 562. No virulence loci, including rmpADC/rmpA2, were detected in any isolate.

**TABLE 1:** Number of core genome single nucleotide polymorphisms between *K. pneumoniae* PDR isolates.

Strains (date of isolation)	Kpn-1 M28162 (8/12/22)	Kpn-2 M28195 (8/28/22)	Kpn-3 M28196 (9/01/22)	Kpn-4 M28206 (9/15/22)	Kpn-5 M28413 (10/01/22)
Kpn-1 M28162 (8/12/22)	0	82	38	75	51
Kpn-2 M28195 (8/28/22)	82	0	56	89	63
Kpn-3 M28196 (9/01/22)	38	56	0	45	31
Kpn-4 M28206 (9/15/22)	75	89	45	0	68
Kpn-5 M28413 (10/01/22)	51	63	31	68	0

Abbreviations. PDR: pan-drug resistant. Kpn: *Klebsiella pneumoniae*.

**FIGURE 1:**
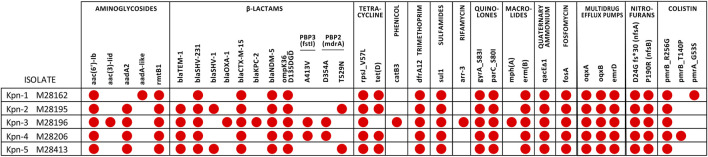
Resistance genes and/or chromosomal mutations in pan-drug resistant isolates obtained by WGS. Red dots indicate the presence of the indicated gene or mutation.

**FIGURE 2:**
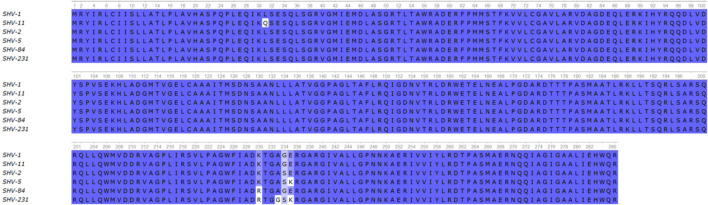
Alignment of the amino acid sequences of selected SHV variants. Conserved residues of the ß-lactamase are denoted in blue while mutations are highlighted in white.

WGS confirmed the presence of *bla*_NDM-5_ and *bla*_CTX-M-15_ in all strains, and *bla*_KPC-2_ in Kpn-3 (Figure 1). A novel allelic variant of the *bla*_SHV_ gene (*bla*_SHV-231_) was present in all isolates (Accession No. OP951208). *bla*_SHV-231_ exhibited two non-synonymous substitutions: K230R and A233G compared to *bla*_SHV-5_ (Figure 2), and its upstream promoter sequence harbored a single-nucleotide change in the +10 region. In Kpn-1, *bla*_SHV-231_ was located on a 150 kb chromosomal contig, as indicated by its genomic context, which included housekeeping and regulatory genes such as *ftrA, duf4177, aac, lacI, lacZ, emrB, ermD, glpR, adh, fruK, fbaA, ptsH*, and *gntP*. In the remaining strains, *bla*_SHV-231_ was found on shorter contigs (3,214–3,995 bp), whose gene surroundings similarly support a chromosomal location. Resistance to aztreonam-avibactam was not transferable in biparental conjugation assays after 48 h of incubation, supporting the chromosomal nature of this resistance determinant. Two isolates (Kpn-2 and Kpn-5) harbored a chromosomal gene duplication event, carrying both, *bla*_SHV-231_ and the ancestral *bla*_SHV-1_ allele.

All isolates possessed IncFIB(pQil), ColRNAI, and Col (pHAD28) plasmid replicons. IncFII was present in all strains except Kpn-1, and IncM1 was exclusive to Kpn-3. IncFIB(K) was found in Kpn-2 and Kpn-3. Although *bla*_KPC-2_ was not co-located with a plasmid replicon in the assembly, the exclusive presence of an IncM1 plasmid in Kpn-3—currently associated with *bla*_KPC_ dissemination in Argentina—suggests a plasmid-borne origin.

All isolates contained multiple resistance genes spanning various antibiotic families. Notably, modifications in outer membrane porins, such as OmpK36/OmpC, and the colistin-associated *pmrB* gene mutation (R256G and T246A) were present (Figure 1). The isolates displayed no difference in iron metabolism *cirA, fepA, fhuA, iroN, btuB, ExbB, ExbD, TonB* genes. Seven unique mutations, including one in *rnpA*, an RNA degradation protein, were found in the cefiderocol-resistant isolate Kpn-5.

Core genome analysis revealed polymorphisms in PBP3 (A413V) for Kpn-3 and Kpn-4 and PBP2 (D354A in Kpn-3 and Kpn-4, T529N in Kpn-2 and Kpn-5). The A413 V mutation in PBP3 is predicted to alter the structural loop adjacent to the active site, potentially diminishing the binding affinity of aztreonam (The PyMOL Molecular Graphics System, Version 3.0 Schrödinger, LLC). No evidence of the amino acid insertions in the β-lactam binding pocket of PBP3 commonly associated with impaired affinity for aztreonam were observed (Periasamy et al., 2020; Sato et al., 2020).

### 3.3 Evaluation of alternatives treatment regimens: synergistic combinations for PDR infections

**FIGURE 3:**
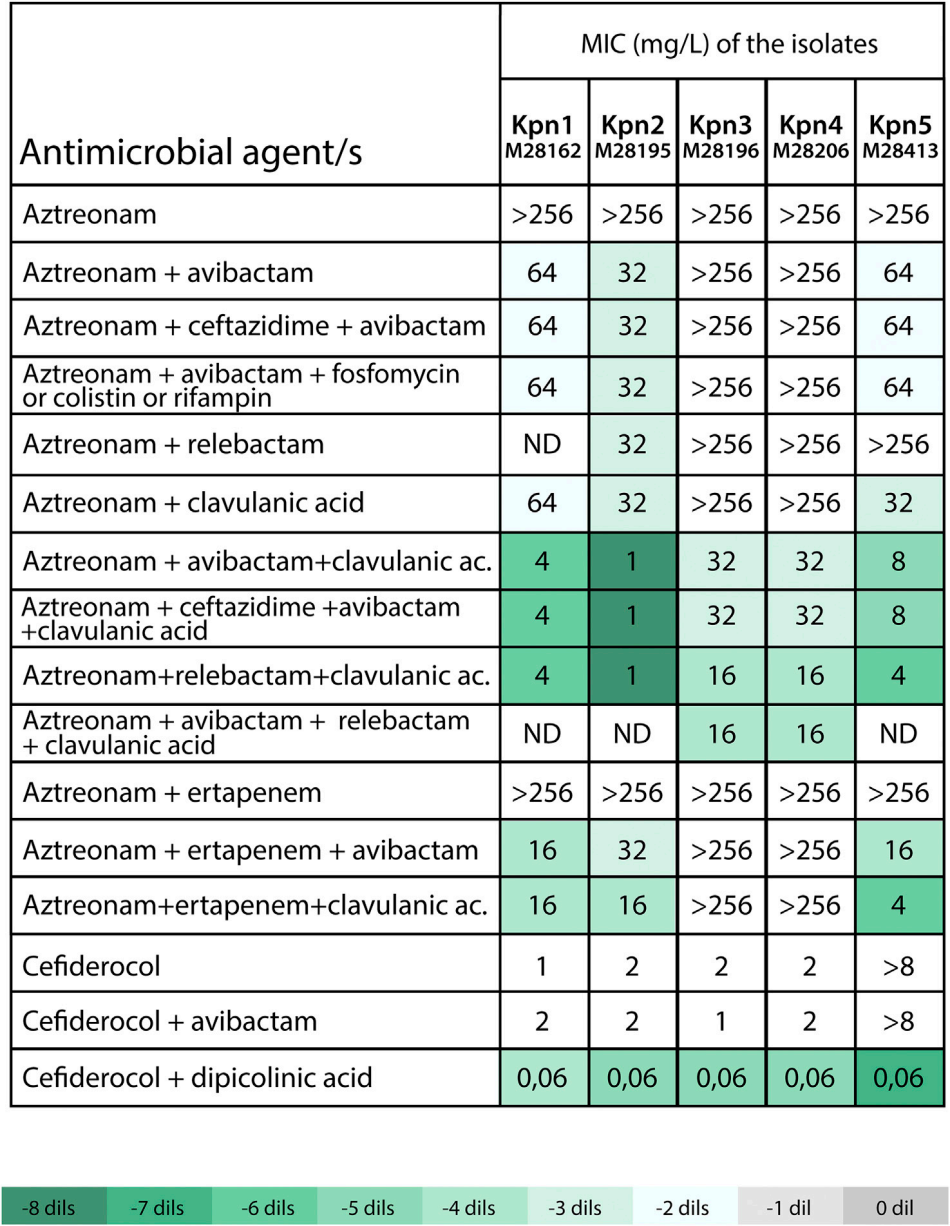
Susceptibility to selected antimicrobial agents alone or in combinations against PDR *K. pneumoniae isolates*. Avibactam, relebactam, clavulanic acid and rifampin were tested at fixed concentration of 4 mg/L, ceftazidime at fixed concentration of 8 mg/L, ertapenem and fosfomycin at fixed concentration of 10 mg/L and dipicolinic acid at fixed concentration of 1,000 µM. ND: not determined. Abbreviations. XDR: pan-drug resistant. Kpn: *Klebsiella pneumoniae.* The color-coded scale in the table indicates the log_2_ (dils.) difference between MICs for β-lactam with the inhibitor in relation to MICs for β-lactam alone (aztreonam or cefiderocol) for each assessed clinical strain, following the provided reference in Table.

Due to the lack of effective standard therapies, alternative regimens were investigated to provide compassionate-use treatments for the affected patients. Aztreonam paired with avibactam, relebactam, or clavulanic acid showed limited effectiveness in isolates Kpn-1, Kpn-2, and Kpn-5 (MICs 32–64 mg/L), while Kpn-3 and Kpn-4 maintained resistance at MICs >256 mg/L (Figure 3). The rationale for drug selection in this compassionate treatment was based on a strategic combination of β-lactamase inhibitors with aztreonam to overcome complex resistance mechanisms. Avibactam was employed primarily to inhibit additional β-lactamases known to compromise the activity of monobactams, such as *bla*_CTX-M_ (present in Kpn-1 to Kpn-5) and *bla*_KPC_ (identified in Kpn-3). To address the presence of an ESBL variant with mutations suggesting an inhibitor resistance profile, such as *bla*_SHV-231_, a range of combinations were evaluated—including both legacy and newer-generation β-lactamase inhibitors, as well as non-classical options like rifampin, fosfomycin or colistin, which may enhance aztreonam activity through synergistic effects. The combination of clavulanate and avibactam outperformed other combinations with aztreonam, including dual β-lactam, fosfomycin, rifampicin, or colistin therapies. The combination of these two inhibitors lowered aztreonam MICs to 1–8 mg/L for all isolates, except Kpn-2 and Kpn-3 (MICs 32 mg/L), and resulted in successful treatment (Figure 3).

Kpn-5 was the only isolate resistant to cefiderocol. Interestingly, *in vitro* treatment with dipicolinic acid restored susceptibility to cefiderocol, highlighting the role of NDM-5 in this resistance phenotype. To further investigate the mechanism underlying this resistance, we conducted an *in silico* analysis of the *bla*_NDM-5_ promoter region, alongside a comparative analysis of the relative gene copies of the *bla*_NDM-5_ contig versus the constitutive gene *gyrA*. The promoter region of *bla*_NDM-5_ was found to be conserved and identical to previously reported sequences (Li et al., 2022). However, gene copies analysis revealed a stark difference between *bla*_NDM-5_ and other isolates, with the copies of *bla*_NDM-5_ in Kpn-5 being significantly higher (5.0 vs. 0.3–1.06 in other isolates) (Supplementary Table S1). This finding reinforces the hypothesis that overexpression of *bla*_NDM-5_ is a key driver of cefiderocol resistance in the absence of mutations in the previously mentioned iron transporter genes. This overexpression is also likely responsible for the high level of resistance to cefepime-zidebactam observed in Kpn-5.

### 3.4 SHV-231 β-lactamase is responsible for the decreased susceptibility to aztreonam-avibactam

**FIGURE 4:**
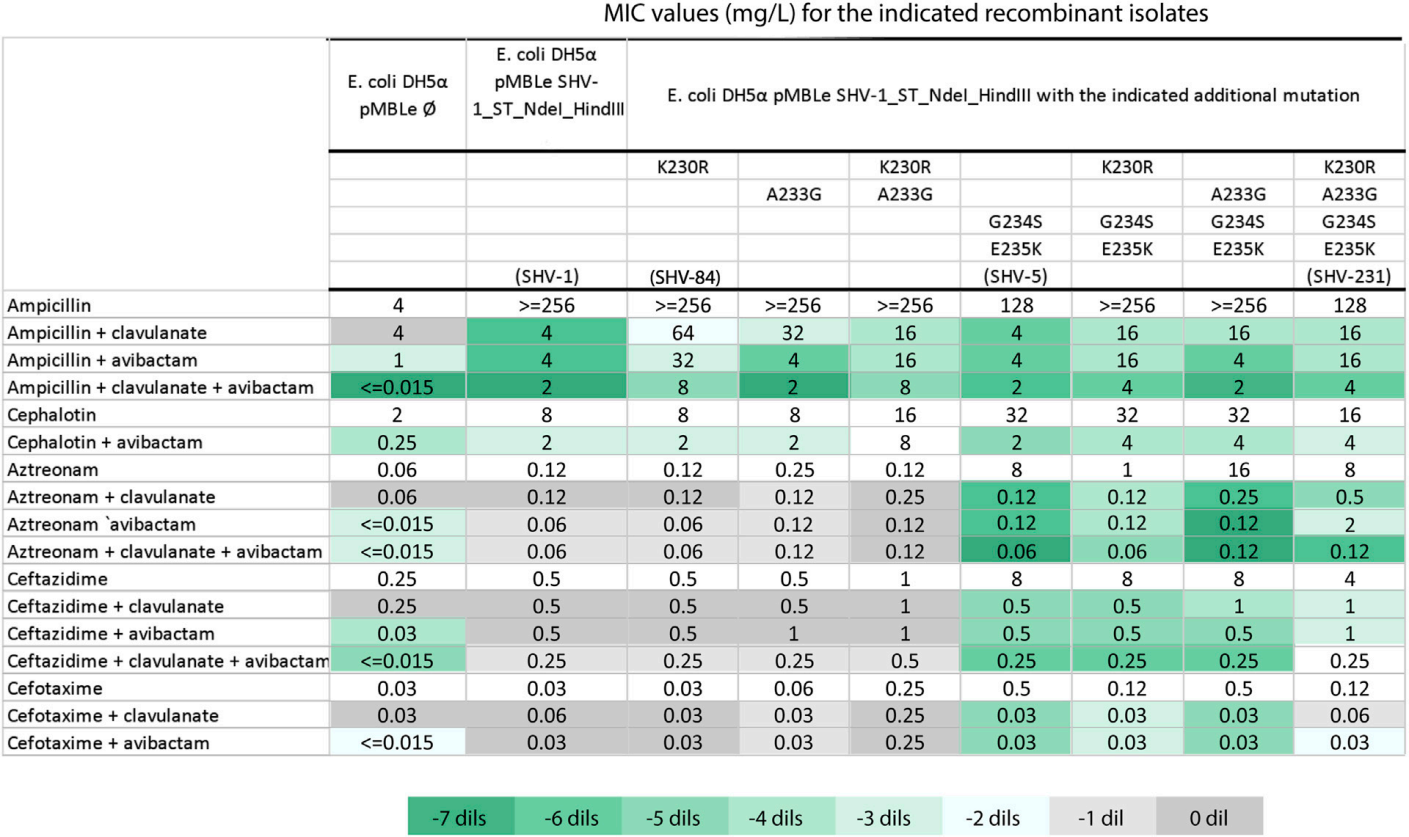
Susceptibility testing of recombinant *E. coli* strains Minimum Inhibitory Concentrations (MICs) were determined for *E. coli* DH5α recombinants carrying either the empty pMBL plasmid or *bla*_SHV-1_ with the specified mutations. The mutation combinations resulting in *bla*_SHV-1_, *bla*_SHV-5_, *bla*_SHV-84_ and *bla*_SHV-231_ are denoted in parentheses. Clavulanate and avibactam were held at a fixed final concentration of 4 mg/L. The color-coded scale in the table indicates the log_2_ difference (dils.) between MICs for β-lactam with the inhibitor in relation to MICs for β-lactam alone for each assessed recombinant strain, following the provided reference in Table.

Since aztreonam resistance has been also associated with the co-production of other ESBLs (Mendes et al., 2021; Findlay et al., 2023) we assessed the potential impact of the *bla*_SHV-231_ variant on the inhibitor resistance phenotype. In follow-up experiments to validate our hypothesis and the clinical response, we cloned the *bla*_SHV-231_ gene into an expression vector in *E. coli*, as well as different combinations of the substitutions present in *bla*_SHV-231_ to dissect their contributions to the resistance phenotype and determined the susceptibility profile of the different variants (Figure 4). *bla*_SHV-231_ carries the two non-synonymous mutations characteristic of *bla*_SHV-5_ (G234S and E235K) responsible of an extended-spectrum phenotype (Billot-Klein et al., 1990). The alteration at K230 stands out because is a highly conserved residue (Dubois et al., 2008; Papp-Wallace et al., 2015). The K230R substitution (*bla*_SHV-84_) not only reduced susceptibility to clavulanic acid, as previously reported (Manageiro et al., 2010) but presented an expanded inhibitor resistance profile that also included avibactam (Figure 4). Substitution A233G alone, instead, affected inhibition by clavulanic acid but not avibactam. The inclusion of K230R in *bla*_SHV-5_ made it more susceptible to aztreonam (MIC 1 mg/L vs. 8 mg/L for *bla*_SHV-5_), but did not affect ceftazidime resistance (MIC 8 mg/L). Instead, addition of both A233G and K230R into *bla*_SHV-5_ (i.e., *bla*_SHV-231_) led to a recovery of aztreonam resistance (MIC 8 mg/L) and a reduced susceptibility to inhibition by avibactam, with only a two-fold decrease in aztreonam-avibactam MIC compared to a six-fold decrease for *bla*_SHV-5_. Notably, the protective efficacy of all tested inhibitors for aztreonam diminished in *bla*_SHV-231_, despite avibactam was more adversely impacted than clavulanic acid. When both inhibitors were evaluated together, the MIC of aztreonam against *bla*_SHV-231_ reached levels comparable to those observed for the *bla*_SHV-5_ allele (Figure 4).

## 4 Discussion

The increasing incidence of PDR Gram-negative bacilli poses a significant clinical challenge, particularly when involving immunocompromised patients (Zowawi et al., 2015; Avgoulea et al., 2018; Lázaro-Perona et al., 2018; Longo et al., 2019). Here we report the spread of PDR NDM-producing *K. pneumoniae* isolates in Buenos Aires. Since its detection in Argentina in 2014, NDM has become endemic, with the COVID-19 pandemic exacerbating resistance, particularly through dual carbapenemase producers (Pasteran et al., 2014; Faccone et al., 2023; Echegorry et al., 2024).

Despite *in vitro* susceptibility to cefiderocol in four of the five isolates, this antibiotic was not considered clinically actionable, as it is not available in Argentina. While the Latin American consensus definition of PDR (Jiménez Pearson et al., 2019) reflects regional realities, alternative frameworks—such as the European consensus by Magiorakos et al. (2012)—define PDR as non-susceptibility to all agents across all antimicrobial categories. In our view, relying solely on definitions that presume universal drug availability may inadvertently exclude the clinical realities faced by low and middle-income countries. A more inclusive and context-sensitive approach to defining PDR may be warranted, ideally through consensus processes that involve equitable participation of all member states.

All five clinical isolates were identified as belonging to Clade II of the high-risk clonal group CG258, a major *K. pneumoniae* lineage known for acquiring carbapenemase genes and contributing to global dissemination. Historically, Clade II has been closely associated with the spread of *bla*_KPC_-producing strains in North and South America (Chen et al., 2014; Lai et al., 2019). Supporting this, a recent multicenter study in Argentina reported *K. pneumoniae* as the predominant host of *bla*_NDM_, with 70% of cases linked to CG258 (Echegorry et al., 2024).

The precise origin of these PDR strains remains elusive. Several factors indicate the local emergence of PDR microorganisms at the institutional level as the index case had previously undergone treatment with ceftazidime-avibactam plus aztreonam in the months leading up to the recovery from the initial strain. Regrettably, surveillance practices, limited to carbapenemase detection, may have enabled the spread of these strains. Following identification of these strains displaying aztreonam-avibactam resistance, the ultimate line drug in Argentina, measures taken for infection control were reinforced, leading to containment of the PDR strains by the end of June 2023.

All five *K. pneumoniae* strains contained between 22 (Kpn-4) and 29 (Kpn-2) antibiotic resistance genes across different drug families. All isolates carried multiple β-lactamases, including *bla*_NDM-5_ and *bla*_CTX-M-15_, aminoglycoside methylases and other modifying enzymes and several core genome target mutations. Amino acid substitutions R256G and T246A in *PmrB* validated colistin resistance and have been linked with CG258 in South America (Longo et al., 2019). The V57I mutation in *rpsJ*, associated with tigecycline resistance outside the AcrAB-TolC system, is also linked to this clonal complex (Villa et al., 2014). The loss of major porin OpmK36 could have increased MICs to β-lactams and generated cross-resistance to other drugs (Tsai et al., 2011). This phenomenon has been frequently observed in the CG258, which multiples reports across South America (David et al., 2019).

### 4.1 Clinical management through compassionate treatment

In the absence of dedicated trials for treating PDR infections, clinical management relies on case reports and limited studies, often outside the renal transplant context (Karakonstantis et al., 2019; Sivasankar et al., 2023). Recognizing the urgent need for treatment alternatives, we exploited genomic insights to guide compassionate therapy. Given the abundance of resistance genes that confer resistance to conventional drugs, we focused on aztreonam by using locally available legacy and newer-generation β-lactamase inhibitors. Patients received treatments involving maximum doses of aztreonam, ceftazidime-avibactam, and oral amoxicillin-clavulanate, leading to clinical cure. Although the triple regimen exhibited higher MIC values, therapeutic levels in the urine were achievable (Ramsey and MacGowan, 2016). However, one patient required an extended treatment course to prevent relapse.

### 4.2 Genomic path to pan-β-resistance

The observed resistance to aztreonam-avibactam stemmed from mutations in PBP2 and PBP3, and a novel SHV-231 β-lactamase. *bla*_SHV-231_ had two nonsynonymous mutations (K230 R and A233G) compared to *bla*_SHV-5_ (Billot-Klein et al., 1990), maintaining the ESBL phenotype while reducing susceptibility to aztreonam-avibactam, a rare trait in this β-lactamase, potentiated by overexpression due to its promoter region. This evolutionary perspective was anticipated by Winkler and coworkers (Winkler et al., 2015). Using an *E. coli* expression model, we confirmed that the triple combination therapy reduced MIC values in clones harboring *bla*_SHV-231_, although the observed MICs were significantly lower than those detected in the corresponding clinical isolates. This discrepancy is likely attributable to the limited expression of SHV variants in the laboratory host strains. In the clinical isolates, WGS revealed that the *bla*_SHV-231_ was linked to a strong P-S promoter (where the C in the second position of the −10 region is substituted by an A). This mutation has been shown to elicit a 200-fold enhancement of β-lactamase expression, leading to a significant increase in MICs (Leung et al., 1997; Rice et al., 2000; Kamruzzaman et al., 2015). Therefore, we expect high expression levels of *bla*_SHV-231_ in all clinical isolates. Multiple alignments on contemporary *bla*_SHV_ promoter-associated sequences of *K. pneumoniae* strains using the NCBI database, revealed that out of 1,000 cases reviewed, only 139 contained a P-S promoter. On this basis, we propose that the PDR isolates may have been subjected to substantial selection pressure—potentially driven by multiple prior treatment regimens—leading to the observed promoter mutation. We therefore attribute the pan-β-lactam resistant phenotype to the overexpression of *bla*_SHV-231_ combined with the presence of NDM.

Isolates Kpn-3 and Kpn-4 showed higher resistance to aztreonam-avibactam due to PBP3 and PBP2 mutations. PBP3 lacks the canonical YRIK, YRIM, and YRIP insertions, displaying only the A413V substitution, previously reported in combination with these insertions (Periasamy et al., 2020; Sato et al., 2020). Residue A413 is located within a conserved amino acid loop (positions 402–420) shared across multiple classes of PBPs and is positioned opposite the active site. The substitution of alanine with a bulkier valine—bearing an additional methyl group oriented toward the active site—may introduce steric hindrance that interferes with substrate binding, contributing to ceftazidime and aztreonam resistance (Sauvage et al., 2014; Zhang et al., 2017). PBP2 substitutions, particularly D354A, correlated with high resistance levels to ceftazidime-avibactam (Livermore et al., 2018), even after MBL inhibition, since MICs remained at 32 mg/L. This suggest a role of PBP2 as a secondary target for diazabicyclooctanes, although the effect of avibactam is weaker than analogs nacubactam and zidebactam (Morinaka et al., 2015; Livermore et al., 2017). This PBP2 alteration might also contribute to higher resistance to cefepime-zidebactam in these two isolates. In summary, we propose that PBP3 and PBP2 mutations with the synergistic effect of the overexpression of *bla*_SHV-231_, hindered the resensitization of aztreonam even after the introduction of multiple inhibitors in Kpn-3 and Kpn-4. These mutations can be attributed to prior exposure to aztreonam plus ceftazidime-avibactam of these patients. Moreover, patient #4 underwent two previous treatment regimens with this combination.

All five *K. pneumoniae* isolates shared core plasmid replicons—IncFIB(pQil), ColRNAI, and Col (pHAD28)—commonly found in high-risk, multidrug-resistant clones. IncFII, present in four isolates, aligns with its known role in spreading ESBL and carbapenemase genes, particularly in CG258 lineages (Rozwandowicz et al., 2018). IncM1, detected only in Kpn-3, has been recently linked to carbapenemase mobilization, while IncFIB(K) in Kpn-2 and Kpn-3 may contribute to the convergence of resistance and virulence traits (Martin et al., 2023). These results highlight a conserved plasmid backbone with additional isolate-specific replicons, reflecting local evolution and adaptation.

Four initial isolates displayed susceptibility to cefiderocol, while the latest isolate (Kpn-5) acquired resistance by overexpression of *bla*_NDM-5_ without previous treatment with this drug. This finding reinforces the hypothesis that overexpression of NDM-5 is a key driver of cefiderocol resistance in the absence of mutations in the previously mentioned iron transporter genes (Simner et al., 2023). This overexpression is also likely responsible for the high level of resistance to cefepime-zidebactam observed in Kpn-5.

The emergence of pan-β-lactam resistance poses severe clinical concerns. Most recently approved drugs for Gram-negative bacilli are β-lactams, except eravacycline and plazomicin (World Health Organization, 2024), making this resistance particularly alarming. Of greater concern, we note that these isolates present resistance to not-yet-approved drugs, like cefepime-zidebactam, further limiting the therapeutic options against NDM-producing strains. These cases demonstrate that personalized combination therapies can effectively manage PDR infections.

## 5 Concluding remarks

The pan-β-lactam-resistant phenotype observed in five clinical isolates highlights the adaptability of *K. pneumoniae* to the evolutionary pressures in clinical environments. The emergence of a new SHV variant gaining legacy and newer-generation β-lactamase inhibitors resistance while preserving ESBL activity, enhanced by mutations in PBPs provides a picture of this adaptation. This evolutionary convergence occurs in a CG258 clone, capable of withstanding selective pressures and forging new evolutionary pathways. This genomic adaptability emphasizes the need of comprehensive genomic surveillance to curb the spread high-risk clones. This study emphasizes the importance of timely genomic and phenotypic characterization of locally circulating pathogens to develop more tailored therapeutic strategies.

## Data Availability

The datasets generated for this study can be found in the GenBank database under Bioproject PRJNA1062347 (https://www.ncbi.nlm.nih.gov/bioproject/?term=PRJNA1062347) and the following accession numbers (BioSample Accession): Kpn-1-M28162, JAYMDN000000000 (SAMN39296142); Kpn-2-M28195, SAMN39296143 (JAYMDO000000000); Kpn-3-M28196, SAMN39296144 (JAYMDP000000000); Kpn-4-M28206, SAMN39296145 (JAYMDQ000000000); Kpn-5-M28413, SAMN39296146 (JAYMDR000000000).

## Abbreviations

XDR, extensively drug-resistant (XDR); MBL, metallo-β-lactamase; ESBL, extended-spectrum β-lactamases; PBP, penicillin-binding protein; WGS, Whole-genome sequencing; UTI, urinary tract infection; CG, clonal group.
