# Supplementary material for: From genomics to treatment: overcoming pan-drug-resistant Klebsiella pneumoniae in clinical settings
Source: Front Pharmacol. 2025 May 30;16:1570278. doi: 10.3389/fphar.2025.1570278 (PMC12162686; doi:10.3389/fphar.2025.1570278)
Supplement: Supplementary file 1 [file Supplementaryfile1.docx]

**
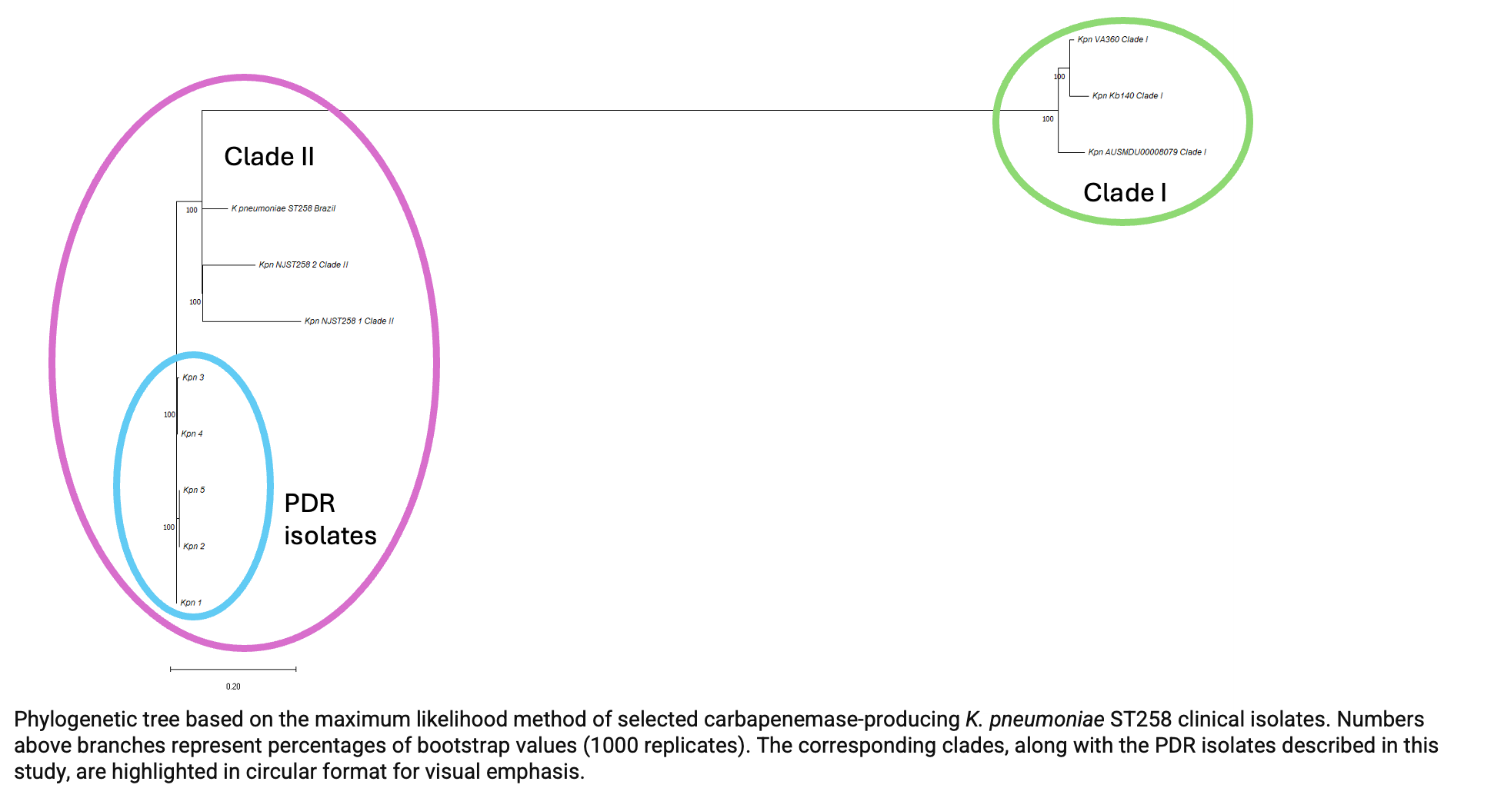
Supplementary Figure 1. Minimum spanning tree based on the allelic profiles of the *Klebsiella pneumoniae* ST258 isolates.**

**Supplementary Table 1 – relative gene copies of *bla*_NDM-5_ versus the constitutive gene *gyrA***

| **Strain ID** | | **Coverage quotient contig NDM / contig gyrA** |
| --- | --- | --- |
| **Kpn-1** | **M28162** | 0,30 |
| **Kpn-2** | **M28195** | 0,4 |
| **Kpn-3** | **M28196** | 1,06 |
| **Kpn-4** | **M28206** | 0,44 |
| **Kpn-5** | **M28413** | 5 |
